# Using Home Visit Notes to Predict Social Needs Among Homebound Older Adults: Lessons From MedStar House Call Program

**DOI:** 10.1093/geroni/igaf122.1469

**Published:** 2025-12-31

**Authors:** Yijung Kim, Valeria Londono, Christine Chow, Lauren Bangerter, Karl De Jonge

**Affiliations:** Medstar Health Research Institute, Columbia, Maryland, United States; Georgetown University School of Medicine, Washington, District of Columbia, United States; Georgetown University School of Medicine, Washington, District of Columbia, United States; Medstar Health, Columbia, Maryland, United States; MedStar Washington Hospital Center, Washington, District of Columbia, United States

## Abstract

Over four million U.S. adults are homebound, facing challenges related to social determinants of health (SDoH). Despite their impact on health outcomes, SDoH data for this population are often underrepresented in electronic health records. To assess prevalence and variability in social needs among homebound older adults, we analyzed unstructured text data from the MedStar Washington Hospital House Call Program, identifying SDoH patterns and evaluating automation using large language models. Our dataset included 1,823 home visit notes from 671 homebound older adults (mean age=88.03; 77% female; 83% Black/African American) written between 2021-2024. Using keyword-based identification, we extracted 702 SDoH-related sentences, manually coding them into nine categories. We examined correlations between identified SDoH patterns and patient characteristics, including demographics, baseline diagnoses, and emergency department (ED) use in the 12 months post-enrollment. To assess scalability, we evaluated SDoH identification using an instruction-tuned large language model (GPT-3.5). Among SDoH categories, excellent care/support was most frequently documented (44%), followed by caregiver burden (21%), social isolation (20%), unsafe environment (11%), lacking transportation (9%), substance abuse (9%), economic instability (7%), poor nutrition (5%), and grief (5%). Correlation analysis revealed excellent care/support was negatively associated with other adverse SDoH factors. In a regression model adjusting for demographics and baseline diagnoses, poor nutrition significantly predicted increased ED utilization. GPT-3.5 demonstrated moderate performance in SDoH identification (precision=0.85, recall=0.74, F1=0.76). Our findings highlight diverse SDoH patterns among homebound older adults. While GPT-3.5 showed promise for automation, we discuss policy and regulatory challenges in implementing the model for clinical use.

